# Fibromyalgia and Myofascial Pain Syndrome-A Dilemma

**Published:** 2009-10

**Authors:** H C Chandola, Arunangshu Chakraborty

**Affiliations:** 1 Professor and Head, Department of Anaesthesiology and Critical Care, M.L.N. Medical College, Allahabad, India; 2 P G Student, Department of Anaesthesiology and Critical Care, M.L.N. Medical College, Allahabad, India

**Keywords:** Myofascial pain, Fibromyalgia, Taut bands, Trigger points

## Abstract

**Summary:**

Pain and fatigue associated to the musculoskeletal system are among the leading causes of patients to visit their physicians and nearly one-third of such patients suffer from fibromyalgia. Fibromyalgia syndrome (FMS) is a chronic debilitating disorder characterized by widespread pain with tenderness in specific areas, leading to fatigue, headache and sleep disorder. Myofascial Pain Syndrome (MPS), is also a localized musculoskeletal pain producing condition whose diagnostic and management criteria differ from FMS but still considered by many only a subtype of FMS. Till date no exact cause has been held responsible for these painful conditions, therefore treatment of these disorders is always a challenge. The therapies are not precise but multimodal including pharmacological and alternative approaches. This article describes the existing knowledge pertaining to these conditions in regard of causative factors diagnosis and management.

## Introduction

Musculoskeletal system is the largest organ system by weight in the human body comprising of more than 400 skeletal muscles^1^. Problems associated with pain or fatigue to this system are among the leading reasons for patients to visit their clinicians^2^,^3^. Majority of these patients fall under the category of either fibromyalgia (FMS) or its subtype myofascial pain syndrome (MPS). Fibromyalgia syndrome (FMS) is a systemic disorder of widespread pain, a consequence of abnormal pain processing within the central nervous system (CNS). As corroborative evidence, recent studies have found increased levels of glutamate, an excitatory neurotransmitter in CNS of fibromyalgia patients^4^. It is one of a number of overlapping functional somatic syndromes which includes chronic idiopathic lower back pain, tension headache, irritable bowel syndrome, chronic fatigue syndrome, disturbed sleep and others.

Fibromyalgia had been included in the tenth revision of the International Statistical Classification of Diseases and Related Health Problems” (ICD-10) along with rheumatism and fibrositis by WHO in 1992 (M 79.0) but is currently classified as a separate entity M 79.7^5^. May 12^th^ has been designated as the International Awareness Day for FMS and other chronic immunological and neurological diseases. Thereby fibromyalgia has emerged from past obscurity and is being recognized with more importance as an “underdiagnosed” but common disease.

One more condition similar to FMS named myofascial pain syndrome (MPS) was described as early as 1843^6^ but debate over its existence as a separate clinical entity from FMS still continues and many consider it only a subtype of FMS. It is true that the diagnostic criteria, clinical features and perhaps the etiopathogenesis of MPS differ from FMS, so the treatment and prognosis^7^. Hans et al (1999) described the differentiating features of MPS from FMS (Table-1).

**Table 1:** How to Distinguish Myofascial Pain Syndrome (MPS) From Fibromyalgia

Feature	Myofascial Pain Syndrome	Fibromyalgia
Trigger/Tender points	Few, localized trigger points	Multiple, generalized tender pts
Musculoskeletal pain	Localized	Generalized
Taut band	Seen	May be seen
Twitch response	Normal	Normal
Referred pain	More frequent	Less frequent
Fatigue	Less frequent	More frequent
Poor sleep	Less frequent	More frequent
Paresthesia	Less frequent	More frequent
Headache	Less frequent	More frequent
Irritable bowel	Less frequent	More frequent
Sensation of swelling	Less frequent	More frequent

Adapted from Hans SC, Harrison P: MPS and TP management, Reg Anesth 1999; 22(1):89–101

The common important feature to both conditions is muscle pain along with the taut or rope like bands in the muscles. In MPS, the painful points in the ‘taut bands’ are called “trigger points” (TP). These points are so precise and painful that on their palpation, patient shows a “jump sign” associated with referred pain. The “tender points” within the sore muscle of fibromyalgia are not associated with jump sign or referred pain.

Pain patterns and identification of TPs may become easier if structural and physiological principles of muscle contractions are well understood.

## Mechanism of contraction of skeletal muscle:

**Fig 1:**
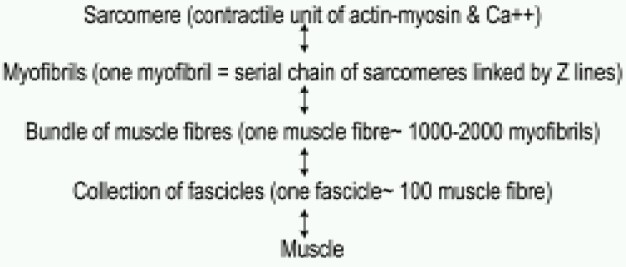
Summary of skeletal muscle structure:

Each skeletal muscle comprises of bundle of fasicles and each fascicle is composed of about 100 muscle fibres. A muscle fibre consist of 1000-2000 myofibrils. Each myofibril is made up of chains of sarcomeres, connected end to end in a serial manner. A sarcomere is a basic contractile unit. Sarcomeres connected by “Z lines” are composed of actin and myosin molecules (Fig 1). Actin and myosin molecules form cross bridges in the presence of ionized calcium (Ca^++^). Actin-myosin bridges remain relaxed till ATP molecules are bound to myosin. Breakdown of ATP to ADP by hydrolysis causes cross-bridging of actin and myosin molecules. Soon ADP molecules leave the myosin causing it to bend which gives a pull on actin and results in shortening of sarcomere. Attachment of another ATP again relaxes the cross bridge to restart the cycle. Repetition of these cycles causes muscle to contract in presence of Ca^++^. Removal of Ca^++^ from the site causes termination of contraction. If additional ATP is not provided because of any reason the cross bridges remain attached and the muscle remains taut or stiff.

Reduced calcium metabolism in the sarcoplasmic reticulum can give rise to a trigger point in a resting muscle similar to a sustained contraction or “tetany”.

Ca^++^ release through the sarcoplasmic reticulum is controlled by release of acetyl choline at the motor end plate. The TPs of MPS are initially involved with motor end plates^8^. This pain can be relieved by stretching back the sarcomere, thus removing the overlap of actin and myosin and restoring the muscle length^9^.

## Factors generating trigger points:

No single factor can be held responsible for the production of TPs. The possible causes are mentioned below.

1. Trauma to:-musculoskeletal system, -intervertebral discs

2. Inflammatory conditions e.g. cholecystitis, appendicitis, gastritis.

3. Myocardial ischemia,

4. Excessive or lack of exercise and malpositions

5. Generalized fatigue, lack of sleep and emotional stress.

6. Hormonal changes as in post menopausal syndrome.

7. Nutritional deficiencies.

8. Intense cooling of body areas … as sleeping in front of A.C.

9. Obesity^10^

10.Use of tobacco^11^

### Types of Trigger Points:

TPs may be one of the following types^12^:

**1. Active TP:** It is a classical TP which is present within a taut band of muscle giving rise to a “Jump sign” on palpation.

**2. Latent TP:** In this case the patient may present a nodular area in a taut band within muscle but does not produce pain on palpation. It is a dormant area that can potentially behave like an active TP later on.

**3. Secondary TP:** It is a hyperirritable point in a muscle that becomes active as a muscular overactivity of another muscle.

**4. Satellite myofascial point:** It is a hyperirritable spot that becomes active because the muscle harbouring it is located within the region of another TP.

### Identification of TPS:

Pain diagrams depicting TPs on human body 13 and some other criteria as essential and confirmatory^14^ have been laid down to identify TPs.

### i Essential Criteria:

- Palpable taut band in muscle

- Spot tenderness of a nodule in band

- Patient's recognition of current pain complaint to pressure on nodule

- Painful limit to stretch motion.

### ii Confirmatory observation:

- Visual or tactile identification of local pathology

- Observation of a local twitch response induced by needle penetration of a tender nodule.

- Altered sensation or pain on pressure on nodule along the area of expected distribution.

- Demonstration of spontaneous electromyographic activity characteristic of active foci in the nodule or band.

### Laboratory Tests:

The pathogenesis of FMS is unknown and there is no single precise test for its diagnosis. Following biochemical changes have been found:

i) Significant rise in proinflammatory cytokines IL-8 and TNF α 15 but no significant changes in IL-4, IL-6, and IL-10 have been reported.

ii) High plasma levels of MCP-1 and eotaxin have been found.^16^

iii) Low serum cortisol due to an adrenocortical deficit has been described^17^

### Treatment of MPS and FMS:

Although many theories have been put forward no clear causative factors responsible for MPS and FMS have been isolated. Association of prolonged static postures, lack of exercise, high body mass index (BMI), sleep disturbance and emotional stress have been found. The treatment at present described for these conditions is therefore multimodal in nature and can be categorized as Pharmacological and Non-pharmacological therapies. The present practice combines non-pharmacological approaches with short term pharmacological therapies for longer lasting and maximal benefits.

### 1.Pharmacological therapies:

Non pharmacological approaches may be common to both conditions but pharmacological managements of MPS and FMS differ.

#### A.MPS:

**i) Trigger point injections:** Injection of TPs with 3% promethazine hydrochloride, 0.5% Procaine or 1% plain lignocaine have been advocated. This therapy is effective when there are only few and precisely located TPs. International Association for the Study of Pain (IASP) recommends some standards and precautions while injecting TPs^18^.

**ii) Spray and stretch with vapocoolant:** The physician uses a heating pad or moist heat on the area for 5-10 min after stretching the affected muscle (around TP). The skin is then sprayed with repeated parallel sweeps of vapocoolant slowly at 10 cm/sec speed and not exceeding two passes over the same area. Some physicians prefer “spray-stretch-spray” sequence^19^. In place of moist heat ice- stroking and coolant like fluorimethane^19^ have also proven useful. Fluori-methane is being replaced by liquid nitrogen or ethylchloride since the former causes damage to the ozone layer^20^.

**iii) Topical analgesics:** Sprays, sport creams and ointments having analgesic properties can be useful to control MPS's pain. Topical application of menthol, peppermint, eucalyptus oil, capsaicin and other herbal preparations can also relieve pain^21^. Capsaicin applied topically degranulates and depletes the substance P store in nerve endings, thereby decreasing pain. Clinical trial of Capsaicin 0.1% (500mcg) hydrogel 2.5 cm diameter patch applied over TPs for cervical myofascial pain is in the second phase.

**iv) Glucosamine and Methylsulfomethane:** When taken orally for a prolonged period they are beneficial owing to their anti-inflammatory and muscle relaxing properties. Many other nutrients e.g. vitamin E, vitamin C, Zinc, Copper and herbal preparations have also been advocated. Recently L-acetyl Carnitine has been found to be effective in fibromyalgia patients. 22

**v) NSAIDS:** These medications are given only for a short period especially in acute stages to reduce pain and inflammation and to enhance relaxation. They have not been proven to increase healing of affected areas. A number of analgesics and anti-inflammatory drugs e.g. aspirin, acetaminophen, ibuprofen are available with their merits and demerits.

**vi) Botulinum toxin (botox)** has been used with mixed results. Injection directly in the TP produces inconsistent effects.^23^ Early reports suggest its use in correcting abnormal biomechanics that incite a myofascial response. 24

### B. Fibromyalgia:

Fibromyalgia shares common underlying neurobiological mechanisms along with physical, cognitive and behavioral co-morbidities. Pain in FMS is supposed to be “central” in origin; the pain relievers like NSAIDS and opioids which are effective on “peripheral” pain are not so effective in this condition. Antidepressants, antiepileptic drugs and a number of neuroactive compounds seem to be more effective in this sort of pain^25^.

i) Oral pregabalin, a Ca++ channel α (2) o subunit ligand with antiepileptic, analgesic and anxiolytic properties has recently been approved in USA for FMS^26^,^27^.

ii) Oral antidepressant drugs like duloxetine^28^ and milnacipran^29^ the combined noradrenaline and serotonin uptake inhibitors are quite effective as pain relievers in FMS. Duloxetine in the dosages of 60-120 mg/day for a long period^28^ and milnacipran 200 mg/day for 27 weeks^30^ appear to be safe, well tolerated and efficacious.

iii) Tropisetron, a 5 HT3 receptor antagonist may also provide significant pain relief but requires i.v route^31^.

iv) Pramipexole, a dopamine (DA_3_) receptor antagonist in the dosages of 4.5 mg/day for weeks also causes improvement in pain, fatigue & global status^32^.

v) Mirtazepine, which blocks α_2_ auto (NA) and heteroreceptors (5HT) is also a promising antidepressant drug^33^ that has been proven useful.

vi) Central muscle relaxants may be combined with analgesics especially in back pain when it is thought to be due to muscle spasm. Cyclobenzaprine, carisoprodol, tizanidine, methocarbamol and metaxalone are the examples of centrally acting muscle relaxants^34^,^35^. Adverse effects e.g. dizziness, drowsiness and drug abuse restricts their use only for a short term.

## Non pharmacological therapies for MPS and FMS:

Due to lack of definitive etiological elucidation and treatment of FMS many alternative approaches have been advocated by pain therapists. The popular approaches have been mentioned below:

i) Choosing correct chair, mattress, and posture to sit or sleep.

ii) Back braces can be used to stabilize the vertebral column or support fatigued muscles.

iii) Traction devices can be used carefully as a temporary pain relief method.

iv) Mechanical massage: Regular massage by the devices available can penetrate deeply through a tapping or percussion action dispersing lactic acid in the soft tissue causing improvement in circulation and relaxation of knotted muscles.

v) Whole body vibration with traditional exercise programme for six weeks was also found to reduce pain and fatigue score.^36^

vi) Chiropractic management combined with aerobic exercises and cognitive behavioral therapy, acupuncture and spa therapy also have strong evidences in their favour^37^.

vii) Yoga: Regular yogic breathing practices, muscle stretching and progressive deep relaxation by “shavasana” are known to have positive effect on FMS.

viii) Ischaemic acupressure or ‘Shiatsu’^38^: In this technique the clinician applies thumb pressure (TP) in a particular manner for 1 minute. In next minute the pressure is increased suddenly aggravating pain and a sensation of “giving away” is felt underneath the thumb in muscle as the pressure is released gradually.

ix) Hot and cold therapies:

– Cold and hot packs: Ice packs can reduce inflammation and pain if applied within 72 hrs of an injury. Ice should not be applied in a single area for more than 20 mins owing to ‘reverse reaction’ phenomenon.

– Hot packs are effective if applied after third day of injury. Moist heat is believed to be better in pain and inflammation improvement.

– Whirlpool and Jacuzzi jet massaging therapy are also examples of moist heat treatment.

– ‘Waon’ (soothing warmth) therapy^39^ employs far infrared ray dry sauna bath at 60° C for 15 min followed by transferring the patients to a room at 26° C covered with blanket for 30 min. Such 2-5 cycles in a week have significant effects on pain reduction.

x) Electrical stimulation: Such devices also prove effective but under medical supervision. Often called “dry needling” the technique of electrical stimulation by a needle passed in to TP has been successfully demonstrated to relieve shoulder and cervical myofascial pain as well as improve microcirculation.^40^

xi) Ultrasound therapy: Sound waves from ultrasound machine are transmitted through sound conducting gel to the tissues. The ultrasound waves break down scar tissue, relax muscle and improve local circulation.

xii) Laser therapy: Short period application of infrared low level 904 nm Ga-As laser therapy have been found to be effective in pain relief and functional ability but its benefit when combined with muscle stretching physiotherapy has been questioned^41^.

The underlying causes of fibromyalgia and myofascial pain syndrome are not yet fully understood and there still remains a controversy about the independent existence of MPS but a high distressing incidence prevails in human population. The acceptance and awareness of these complex disorders has generated the need of new researches in all medical and paramedical fields. At present the combined and collective approaches hold the key to the management of fibromyalgia.
